# Facial Skin Density Enhancement Using Hyaluronic Acid—Based Bioactive Hydrogel: Cannula-Assisted Delivery and Ultrasound Evaluation in a Retrospective Controlled Study

**DOI:** 10.3390/pharmaceutics17050553

**Published:** 2025-04-24

**Authors:** Lidia Majewska, Karolina Dorosz, Jacek Kijowski

**Affiliations:** 1 ESME Clinic, ul. Lwowska 1/U16, 30-548 Kraków, Poland; 2 Biological Sciences Department, University of Chicago, Chicago, IL 60637, USA; k.dorosz@uchicago.edu; 3 Stem Cell Laboratory, Małopolska Centre of Biotechnology, Uniwersytet Jagiellonski w Krakowie Małopolskie, 30-387 Kraków, Poland; jacek.kijowski@uj.edu.pl

**Keywords:** skin regeneration, mesotherapy, non-stabilized hyaluronic acid filler, skin stimulators, skin booster, hyaluronic acid filler, cannula injection, skin biorevitalization, skin bioregeneration, skin ultrasound, skin density, HA fillers

## Abstract

**Background**: Hyaluronic acid (HA)–based bioactive hydrogels have emerged as multifunctional platforms for skin bioregeneration. While traditional mesotherapy using multicomponent substances has been widely practiced for improving skin quality, the time-consuming nature of this approach has led to exploration of alternative delivery methods. **Aims**: This study evaluated the clinical effectiveness of an HA bioactive hydrogel-based bioregeneration system (containing non-stabilized hyaluronic acid and 14 bioactive ingredients) administered via cannula and its impact on facial skin density as assessed by ultrasound imaging. **Methods**: We conducted a retrospective review of data from 20 female patients aged 30–42 years who received a single cannula-delivered injection of a bioactive hyaluronic acid hydrogel (TEOSYAL^®^ Redensity [I]) in the midface region. The formulation combines the structural benefits of hyaluronic acid with the biochemical stimulation provided by amino acids, antioxidants, minerals, and vitamins. Skin density was measured using high-frequency ultrasound at baseline, immediately post-procedure, and at 3–4 weeks follow-up. A control group of seven individuals received no treatment. **Results**: Ultrasound assessments revealed a statistically significant increase in skin density (92.7%, *p* < 0.001) within the treated area compared to no significant changes in the control group. This substantial improvement in dermal architecture demonstrates the efficacy of bioactive hydrogels in stimulating fibroblast function and extracellular matrix regeneration. Patient satisfaction was high, with 85% of patients reporting being satisfied or very satisfied. Side effects were minimal, with minor bruising (10%) and transient swelling (15%). **Conclusions**: Cannula-delivered bioactive hyaluronic acid hydrogel effectively enhances facial skin density with high patient satisfaction and minimal downtime, demonstrating the potential of advanced hydrogel formulations as multifunctional therapeutic platforms that extend beyond traditional applications into aesthetic and regenerative dermatology.

## 1. Introduction

The aging process of the skin is primarily due to the diminished function of dermal fibroblasts and a decrease in their biosynthetic capacity. Regardless of the underlying cause, aged fibroblasts generate insufficient quantities of extracellular matrix (ECM) components necessary for maintaining youthful skin appearance [1].

In aged dermal fibroblasts, a pivotal role is played by the production of matrix metalloproteinases (MMPs) as part of the senescence-associated secretory phenotype (SASP), leading to accelerated collagen breakdown [2]. This combined reduction in collagen production, alongside increased collagen degradation, substantially contributes to loss of skin elasticity, wrinkle formation, and sagging [3].

Mesotherapy products aim to counter and decelerate these age-related skin changes. The development of complex, multicomponent mesotherapy solutions is grounded in the concept that aging skin requires a diverse range of substrates essential for optimal fibroblast function [4].

Varani et al. [5] highlighted that decreased synthesis of type I and III collagen is a hallmark of chronologically aging skin. Studies demonstrated that fibroblasts from younger individuals (aged 18–29) produced higher levels of type I procollagen in vitro than those from older individuals (aged 80+). Additionally, comparisons between young and elderly skin samples revealed higher fibroblast adhesion to collagen fibers and greater cell spreading in young skin, suggesting that fibroblasts in older skin experience reduced mechanical stimulation. These findings indicate that chronologically aging skin is influenced by two parallel processes: the aging of fibroblasts themselves and reduced mechanical stimulation of these cells.

Research indicates that dysfunctional fibroblasts in aging skin still retain the potential for functional activation, suggesting they could serve as effective targets for anti-aging treatments [6]. Prikhnenko [4] argues that a deeper understanding of skin aging mechanisms can aid in identifying biomolecules capable of enhancing or sustaining fibroblast function, thereby promoting the biosynthesis of essential extracellular matrix components.

Recent developments in aesthetic and regenerative dermatology have embraced the concept of bioactive hydrogel-based systems as multifunctional platforms for skin bioregeneration, extending far beyond traditional wound care. Among these, hyaluronic acid (HA)-based hydrogels have gained particular attention due to their intrinsic biocompatibility, biodegradability, hydrophilicity, and non-toxic profile. HA, a major component of the native extracellular matrix (ECM), plays a pivotal role in cell signaling during morphogenesis, inflammation, and tissue repair, making it an ideal biomaterial for applications in both regenerative and aesthetic medicine [7,8].

Once crosslinked into hydrogel form, HA can be transformed into elastic, injectable matrices that serve as carriers for bioactive agents, stem cells, and therapeutic compounds. These hydrogels not only provide mechanical support but actively influence cellular behavior, including fibroblast proliferation, ECM biosynthesis, and even stem cell differentiation—a key factor in skin tissue engineering and rejuvenation strategies. Compared to other natural or synthetic polymers, HA-based hydrogels exhibit superior biofunctionality, making them highly effective in restoring dermal structure and function in aged or photo-damaged skin [9]. In this context, “non-stabilized or non-cross-linked hyaluronic acid filler” refers to HA that has not undergone extensive chemical cross-linking, resulting in a product that maintains the natural bioactivity of HA while providing temporary volumizing and hydrating effects. Unlike highly cross-linked HA fillers designed for structural support and longer duration, non-stabilized HA prioritizes biocompatibility and integration with the skin’s natural processes.

When formulated with additional biomolecules—such as amino acids, antioxidants, vitamins, and trace elements—these multicomponent HA hydrogels evolve into bioactive therapeutic platforms. Products like Teosyal Redensity 1 reflect this multifunctional approach, offering not just structural replenishment but also biochemical stimulation of senescent fibroblasts, improved hydration, protection against oxidative stress, and enhanced collagen synthesis. This positions HA-based hydrogels at the forefront of modern bioregenerative aesthetics [10].

As such, the paradigm of bioactive hydrogels as multifunctional dressing materials extends naturally into anti-aging and skin-regenerative therapies, bridging the gap between materials science and clinical dermatology.

One frequently used multicomponent HA gel is Teosyal Redensity 1 (TEOSYAL^®^ Redensity [I], TEOXANE Laboratories, Switzerland). Teosyal Redensity 1’s formulation is designed to address skin aging comprehensively.

Teosyal Redensity 1 stands out for its unique composition, combining 15 mg/mL of hyaluronic acid with 0.3% lidocaine and 14 bioactive ingredients, including essential amino acids, antioxidants, minerals, and vitamin B6, which support skin regeneration processes and enhance elasticity and hydration.

The amino acids (arginine, glycine, leucine, isoleucine, valine, lysine, threonine, and proline) contribute to collagen synthesis, supporting skin structure; antioxidants (glutathione, alpha-lipoic acid, and N-acetyl-L-cysteine) help combat oxidative stress, protecting skin cells; minerals such as zinc acetate and copper sulfate aid in cellular processes vital for skin health; and vitamin B6 plays a role in metabolism, assisting in overall skin rejuvenation. The product is available in either a single blister containing a 3 mL pre-filled sterile syringe with two 30 G 1/2 inch needles, or as two blisters, each holding a 1 mL pre-filled sterile syringe, also equipped with 30 G 1/2 inch needles.

In vitro studies [11] indicate that following injection, fibroblasts treated with this product show a substantial increase in both synthetic and proliferative activity. Additionally, these fibroblasts exhibit an enhanced ability to withstand oxidative stress [12].

## 2. Objectives

This study aimed to evaluate the clinical effectiveness of multicomponent bioregeneration injections administered via cannula and their impact on facial skin density, as assessed by ultrasound imaging. Additionally, this work serves as a proof of concept to explore the potential of using cannulas instead of traditional mesotherapy techniques, focusing on the outcomes of a single session.

## 3. Material and Methods

A retrospective analysis was conducted on 20 patients treated in our practice between October 2021 and June 2022 using the Teosyal Redensity 1 (Teoxane, Switzerland) injection product. All patients signed consent forms for the use of this biorevitalization product.

The retrospective analysis was conducted in compliance with the study protocol and in alignment with the Declaration of Helsinki, along with adherence to local laws and regulations.

All participants consented to the anonymous analysis of data obtained during treatment, and three individuals agreed to have their images published, with the appropriate documents obtained for this.

The analysis included data from 20 female patients aged 30–42 years (the mean age of participants in the intervention group was 36.2 years ± 2.8 years) with Fitzpatrick skin phototypes I, II, and III and a photodamage level of 1–2 on the Glogau scale.

The exclusion criteria for the procedure included the following: any filler injections within the past year, recent chemical peels (within the past three months), and a history of allergies or anaphylactic reactions. Patients with known hypersensitivity to hyaluronic acid (HA), lidocaine, amide-type local anesthetics, or any components of the product were also excluded. Additional exclusion criteria were as follows: smoking, previous facial laser treatments within the past year, and surgical facelifts within two years prior to the study. The study also excluded patients with a tendency to develop hypertrophic scars or skin inflammation, disorders related to wound healing or coagulation, and those using aspirin or anti-inflammatory medication within seven days prior to treatment. Finally, patients with active skin diseases, dermatoses, autoimmune disorders, chronic or acute systemic illnesses, and those using any topical or systemic therapies that could impact the treatment results were not included.

The procedure involved a single subcutaneous injection (into the superficial dermis) of 3 mL of product in the midface area using a cannula (Steriglide, 25G, 50 mm, TSK, Japan) from one entry point on each side of the face. This specific cannula was selected for its optimal balance of flexibility and precision, allowing for smooth, atraumatic delivery of the product through a single entry point while maintaining precise distribution in the target tissue planes.

Two follow-up visits were also performed: the first visit took place immediately before the initial injection, and the second visit occurred 3–4 weeks after the injection session, during which ultrasound skin scans of the face were conducted. Each patient underwent only one treatment.

**Figure 1:**
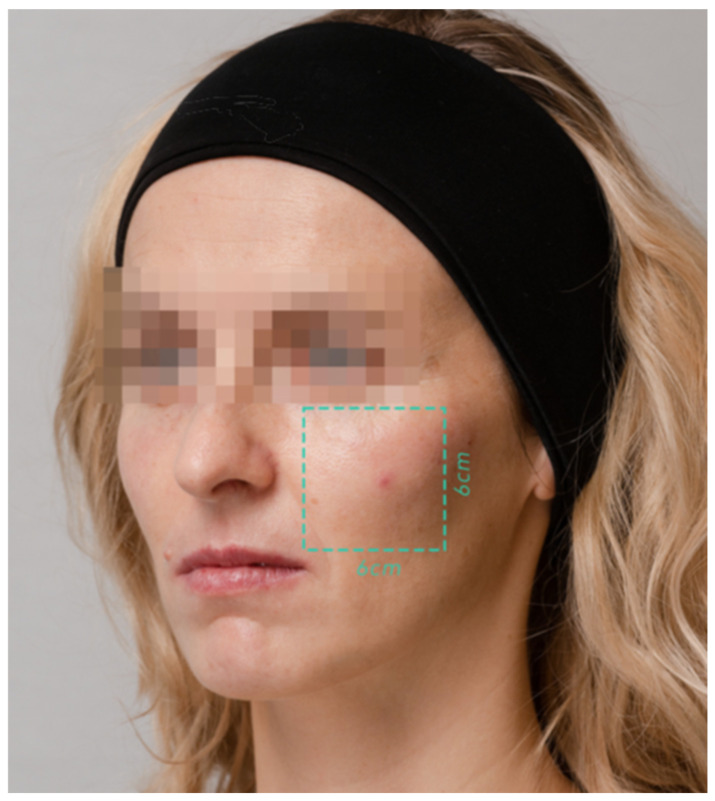
Area 6 × 6 cm marked for injections and ultrasound measurements.

Following skin disinfection, a single entry point was created approximately 3 cm from the porion using a 23 G needle, through which a blunt-tipped 25 G, 50 mm cannula was introduced in a downward direction. The procedure involved a single subdermal injection of the product (Teosyal Redensity I, Teoxane, Switzerland), with a total volume of 1.5 mL per side. The product was administered using a fan-like technique within the superficial subdermal plane, targeting the midface region. The injections were distributed evenly within the designated area, marked using a 6 × 6 cm template (Figure 1), ensuring precise coverage and procedural accuracy.

**Figure 2:**
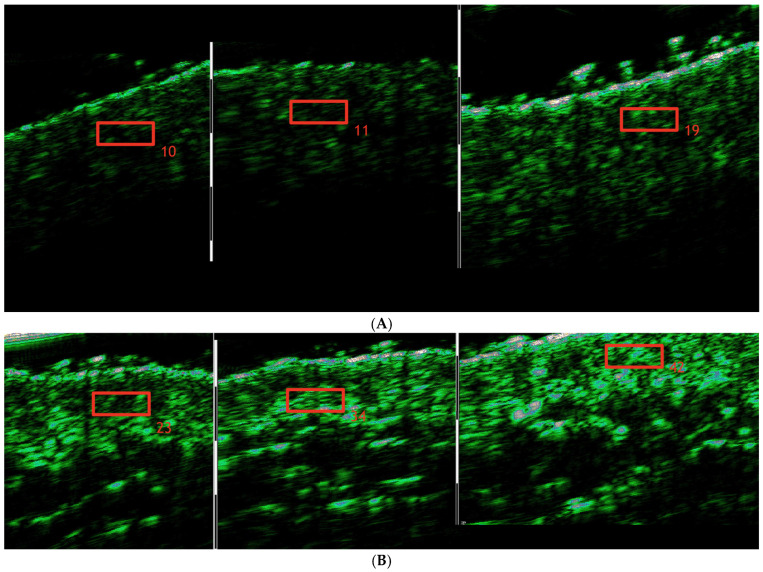
Skin density increase in the cheek area during treatment regimen before the treatment/immediately after treatment/3 weeks after treatment completion. (**A**). Patient #1 (**B**). Patient #2.

Skin density measurements were conducted in the marked region with the DUB^®^ SkinScanner (DUB SkinScanner75, probe: 33–38 MHz, tpm GmbH, Plymouth, USA), a high-resolution ultrasound device for non-invasive skin analysis, providing clear imaging for assessing the dermis and epidermis (Figure 2). The measurement spot for ultrasound scans was marked using a non-permanent skin marker within a predefined 6 × 6 cm region. This area was measured consistently at each of the three time points to ensure repeatability.

A total of three skin density measurements were taken in the designated area: a reference measurement, a measurement immediately after the procedure, and a follow-up measurement after 3 weeks.

In the control group, consisting of seven women aged 30–43 years (the mean age was 35.9 years ± 2.5 years), skin density was measured only in the midface area (using the same 6 cm × 6 cm measurement spot) during the consultation visit and again after 3–4 weeks. No medical intervention was performed on these individuals. This setup was intended to serve as a baseline for understanding natural variations in skin density over the study period. By comparing the results from the intervention group to the control group, the study aimed to distinguish changes caused by the treatment from those due to natural skin regeneration processes or measurement variability.

## 4. Statistical Analysis

The statistical analysis was conducted using the STATISTICA 13.5 PL software package. For each data point, descriptive statistics—including the mean, standard deviation, minimum, and maximum—were computed. To assess changes over time, repeated measures analysis of variance (ANOVA) was applied. Where significant effects were observed, further pairwise comparisons were conducted using the Tukey post-hoc test. Additionally, power analysis and effect size were calculated to evaluate the robustness of the findings. For comparing two related measurements, the paired t-test was used. A probability level of *p* < 0.05 was set as the threshold for statistical significance across all tests.

## 5. Results

**Figure 3:**
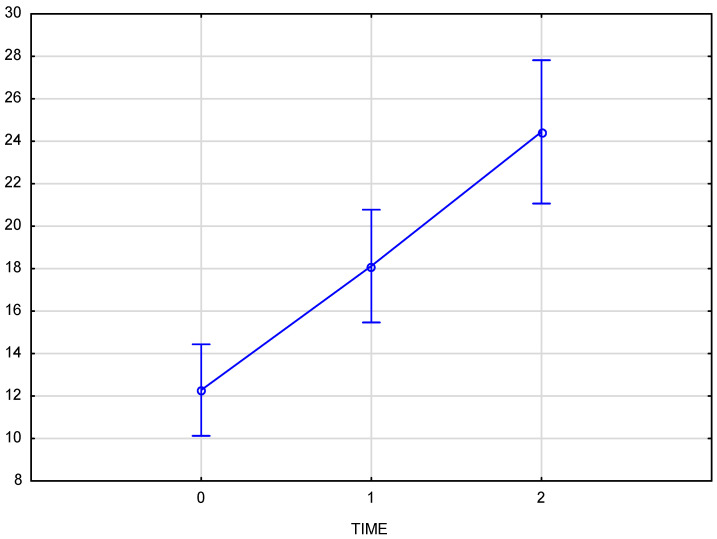
Skin density on the right side of the face, measured at baseline, immediately after injection, and three weeks post-procedure.

Skin density measurements for the left and right sides of the face were analyzed independently. The chart (Figure 3) shows the results for the right side of the face, considering time points (reference measurement (0), measurement immediately after the procedure (1), and follow-up measurement after 3 weeks (2)). The Tukey post-hoc test revealed a significant increase in tissue density and statistically significant differences in the comparisons of measurement 0–1, 1–2, and also 0–2. The partial Eta-squared effect size is 0.66, indicating a substantial impact of the applied therapy.

**Figure 4:**
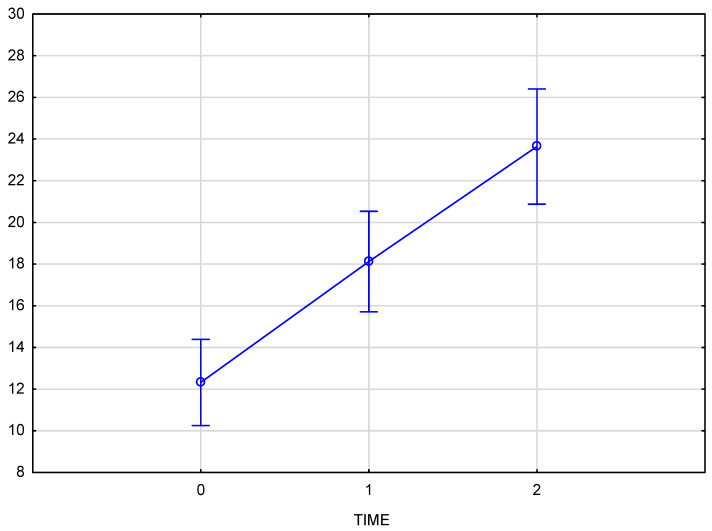
Skin density on the left side of the face, measured at baseline, immediately after injection, and three weeks post-procedure.

The chart (Figure 4) presents skin density results for the left side of the face at three time points: baseline (reference measurement 0), immediately after the procedure (measurement 1), and three weeks post-procedure (measurement 2). The Tukey post-hoc test indicated a significant increase in tissue density, with statistically significant differences across all comparisons: baseline to immediately post-procedure (0–1), immediately post-procedure to follow-up (1–2), and baseline to follow-up (0–2). A partial Eta-squared effect size of 0.76 demonstrates a robust effect of the treatment on skin density.

**Figure 5:**
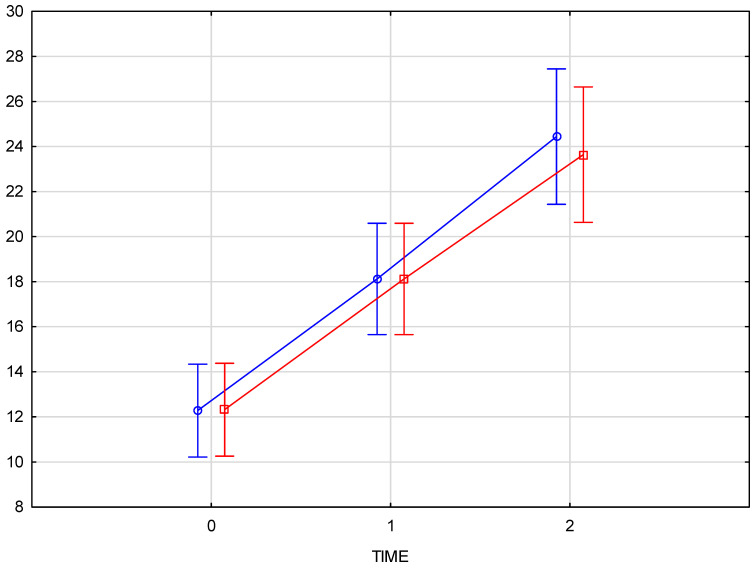
Comparison of skin density results for the right and left sides of the face at baseline, immediately post-injection, and three weeks post-procedure. (blue—right side of the face, red—left side of the face).

The comparison of skin density results for the right and left sides of the face confirms that there is no interaction between the side of injection and changes over time (*p* = 0.82372). This indicates that the progression of changes over time is consistent on both sides of the face, regardless of the injection side (Figure 5).

The arithmetic mean of the measured skin density values at the baseline was 12.05 for the right and 11.95 for the left half of the face, increasing to an average value of 17.8 immediately after the product was administered. The final measurement, taken 3–4 weeks after the procedure, showed an average density increase of 94% for the right side and 92% for the left side of the face. Comparison of skin density between the groups (test group and control group) displays a significant interaction (*p* = 0.000008) between time and group which means that the course of changes over time differs, and that that the progression over time differs significantly from the test group, confirming the therapeutic effect of the applied treatment.

**Figure 6:**
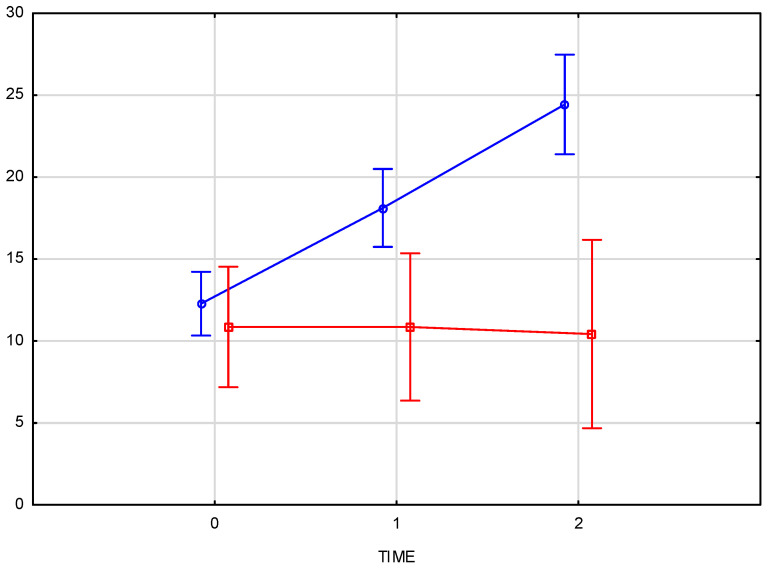
Comparison of skin density changes over time between the test group (L, blue) and the control group (K, red), measured at baseline, immediately after injection, and three weeks post-procedure.

The average increase in skin density of 92.7% was statistically significant (*p* < 0.001), with a strong treatment effect as demonstrated by the high partial Eta-squared values (0.66 for the right side and 0.76 for the left side of the face). The consistency of the effect was evident in the similar increases in skin density on both sides of the face (94% for the right side and 92% for the left side). Comparison of skin density between the groups (test group and control group) displays a significant interaction (*p* = 0.000008) between time and group, confirming the therapeutic effect of the applied treatment. No significant changes in skin density were recorded in the control group at any time point (Figure 6).

**Figure 7:**
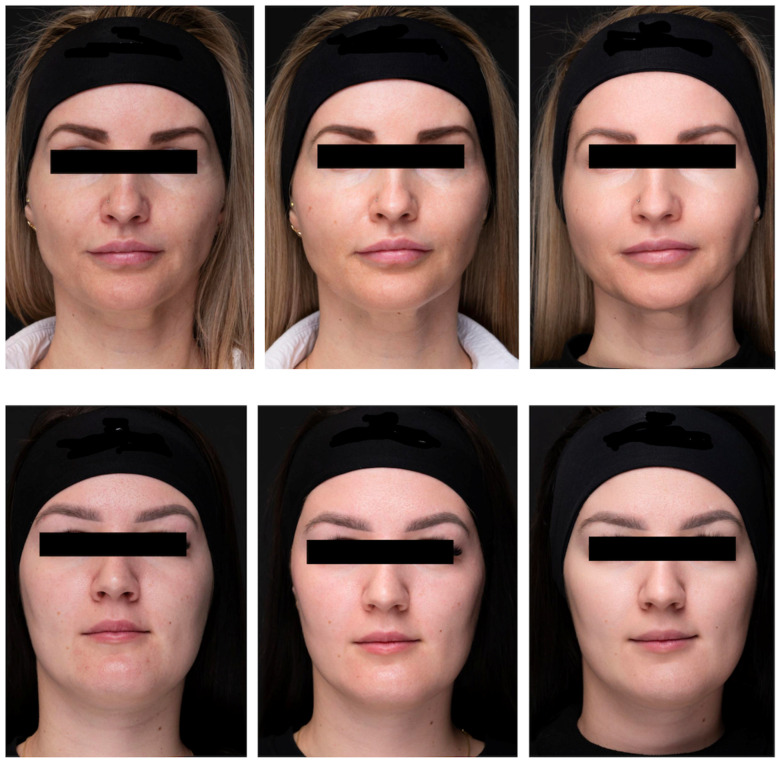
Digital photographs of the patients. (**Top**). Before the treatment/immediately after treatment/3 weeks after treatment completion. (**Bottom**). Before the treatment/immediately after treatment/3 weeks after treatment completion.

Ultrasound measurements and GAIS scores confirmed a positive impact of the multi-component bioregeneration product on skin quality, validating the measurable effects of the cannula-based injection. Digital photo-documentation was also done at baseline, immediately after injection, and three weeks post-procedure (Figure 7).

In terms of satisfaction, 80% (n = 16) would recommend the treatment to friends. Among the participants, 25% (n = 5) were very satisfied, 60% (n = 12) were satisfied, and 15% (n = 3) were not satisfied with the outcome. The overall aesthetic improvement was rated by patients as “improved” with an average GAIS score of 1.7.

The treated area’s aesthetic improvement was evaluated 3–4 weeks post-treatment using the Global Aesthetic Improvement Scale (GAIS), where 0 = worsened appearance, 1 = no visible change, 2 = slightly improved, 3 = noticeably improved, and 4 = significantly improved. Patients also rated their satisfaction with the treatment (options: very satisfied, satisfied, not satisfied) and indicated whether they would recommend the treatment to friends (options: yes or no) (Table 1).

## 6. Side Effects

The incidence of side effects was low, with two (10%) patients experiencing bruising and three (15%) reporting localized swelling, which subsided within 48 h.

## 7. Discussion

Cutaneous aging represents one of the most apparent manifestations of human senescence. Throughout life, skin undergoes progressive alterations. These changes are characterized by the emergence of wrinkles, reduced elasticity, and volume loss. Additional aging signs include sagging, coarsening of texture, and pallor. Photoaging, in contrast, is marked by irregular pigmentation and accentuated wrinkling. At the cellular and molecular strata, the aging of skin encompasses a complex web of influences from exogenous factors, genetic regulations and biochemical pathways. A central cellular disruption in aged skin is the dysregulation of redox homeostasis.

In intrinsic aging, reactive oxygen species (ROS) primarily arise as a byproduct of cellular oxidative metabolism during ATP synthesis from glucose, coupled with mitochondrial inefficiency. In extrinsic or environmental aging, redox disturbance results from factors such as ultraviolet radiation, air pollution, poor nutrition, and tobacco smoke. During the aging trajectory, oxidative stress increases due to both elevated ROS levels and decreased activity of enzymatic and non-enzymatic antioxidant defenses. Beyond these visible structural modifications, aged skin progressively loses its functionality and regenerative capacity. Immunosenescence of the skin further manifests as a diminished capacity to resist infections and an elevated risk of autoimmune and oncogenic pathologies [13].

Hyaluronic acid (HA), a high-molecular-weight glycosaminoglycan, is a key structural and functional component of the skin’s extracellular matrix (ECM), known for its hydrating properties and ability to retain water. In the dermal ECM, HA not only supports structural integrity but also creates a hydrated microenvironment essential for cellular processes. It plays a significant role in maintaining tissue homeostasis, promoting cellular proliferation, and facilitating wound healing.

As skin ages, intrinsic factors such as cellular senescence and extrinsic factors like UV exposure contribute to a decline in endogenous HA synthesis, leading to dehydration, loss of elasticity, and compromised ECM integrity. The exogenous application of HA, particularly in the form of cross-linked fillers, helps counteract these effects by re-establishing dermal volume and hydration. Cross-linked HA is designed to resist rapid degradation, allowing it to remain within the dermal layers longer and providing sustained mechanical support to the ECM.

When administered to aging skin, HA not only hydrates but also induces mechanotransduction pathways that stimulate fibroblast activity. It has been shown to upregulate the Transforming Growth Factor-beta and Connective Tissue Growth Factor (TGF-β/CCN2) axis, promoting the synthesis and maturation of collagen fibers. This pathway is essential in ECM remodeling and cellular responses to mechanical stress, ultimately contributing to improved skin resilience, density, and elasticity [14,15]. Additionally, HA’s viscoelastic properties provide structural support to the skin, reducing wrinkle depth and enhancing tissue firmness, which are critical goals in aesthetic and reparative dermatology.

Quan [6] reached similar conclusions in their research. They found that collagen fragmentation with age reduces fibroblast binding to the ECM and mechanical forces. This leads to fibroblast contraction and diminishes their function, ultimately resulting in decreased collagen production. The authors propose that many age-associated skin changes can be significantly mitigated by reinforcing the structural integrity of the ECM. Injecting HA dermal fillers into the skin of individuals aged 70 and older has been shown to activate fibroblasts to produce type I collagen. Mechanical forces induced by HA injection promote fibroblast elongation and spreading. This response is mediated by TGF-β receptor type II and connective tissue growth factor, both of which play roles in fibroblast mechanotransduction. Strengthened ECM mechanical support has also been observed to stimulate fibroblast proliferation and increase epidermal thickness, collectively contributing to improved skin vitality.

Hyaluronic acid is widely recognized in aesthetic dermatology for its role in skin revitalization. However, the specific applications of HA-based products often lack standardization, limiting their optimal use. In a study [16], researchers reviewed the applications of non-animal stabilized hyaluronic acid (NASHA^®^) gel skin boosters (NSBs) across various anatomical areas and patient demographics, providing detailed recommendations. They suggest that a standardized protocol—consisting of up to three initial sessions followed by regular maintenance treatments—effectively enhances and preserves skin quality over time. Visible improvements are typically noted after the first session, with gradual enhancements in skin texture continuing for up to 12 months with maintenance treatments spaced 4 to 6 months apart. The NASHA gel, upon reaching the dermis, not only rehydrates the skin but also stimulates collagen synthesis, which restores both volume and structural density to aging skin.

In a separate study [17], researchers conducted a retrospective review of medical records from 115 patients who received either non-cross-linked or cross-linked hyaluronic acid (HA) treatments for a range of aesthetic concerns, including wrinkles, skin rejuvenation, age-related skin laxity, scars, and striae. The findings demonstrated a comparable and significant improvement across all these skin issues for both non-cross-linked and cross-linked HA when administered subcutaneously. The overall Global Aesthetic Improvement Scale (GAIS) scores were 1.78 for non-cross-linked HA and 1.6 for cross-linked HA, aligning closely with the results observed in our study.

A separate review [18] assessed the effectiveness of various HA products for skin rejuvenation based on effect duration, injection methods, and adverse event rates. A total of 34 articles were reviewed, including 23 on non-cross-linked hyaluronic acid (HA) and 11 on cross-linked HA. Treatment sessions with non-cross-linked HA ranged from one to six, scheduled weekly to bi-monthly, while cross-linked HA treatments consisted of one to three sessions spaced between 4 and 36 weeks apart. The reviewed studies [16,17,18] highlight the diversity of treatment protocols with non-cross-linked hyaluronic acid (HA), ranging from one to six sessions scheduled weekly to bi-monthly [18], demonstrating that there is no universally established standard for the number of sessions or their frequency. In light of this variability, our study adopted a single-session protocol using a biorevitalizing solution containing non-cross-linked HA as a proof of concept. This approach is equally justified within the spectrum of existing protocols, as it allows for the evaluation of the immediate efficacy and safety of the treatment in a simplified and controlled manner. By employing this protocol, we contribute to the ongoing exploration of effective and flexible treatment regimens, while providing foundational data for further studies to optimize session numbers and intervals based on specific clinical outcomes.

Optimal results depend on selecting the appropriate HA type and ensuring precise placement at the correct dermal depth, which enhances improvement while reducing potential side effects, such as Tyndall effect and skin irregularities. The authors [18] emphasized the importance of adjunctive rejuvenating treatments to further improve aesthetic outcomes, particularly in elderly patients with photodamaged skin. Additionally, maintaining aesthetic improvements requires regular maintenance sessions every few months.

The multicomponent bioactive HA hydrogels represent a promising strategy in skin rejuvenation, particularly in targeting fibroblast senescence and reduced ECM turnover. By providing both hydration and targeted biochemical support, these formulations enhance fibroblast proliferation, collagen synthesis, and resistance to oxidative stress—characteristics essential for restoring skin elasticity and integrity in aged or photo-damaged skin. Therefore, their role as multifunctional dressing materials extends into therapeutic skin revitalization, marking a significant convergence of aesthetic medicine and regenerative biomaterials research.

A review of the existing literature reveals a lack of studies specifically addressing the use of cannulas for the delivery of multicomponent solutions into the skin. While cannulas are commonly used for filler injections [19,20,21,22], their application for multicomponent bioregeneration products remains underexplored. This study, therefore, contributes novel insights into this technique, potentially filling an important gap in dermatological and aesthetic research. Future investigations could further delineate the advantages of this approach, particularly in comparison to traditional needle-based methods.

This work represents a proof of concept to demonstrate the feasibility and preliminary efficacy of using cannulas for the delivery of multicomponent bioregeneration products. Unlike traditional mesotherapy, which involves multiple sessions and frequent punctures, this study employed a single session with cannula injections. Despite the limited intervention, the results showed a significant increase in skin density, highlighting the potential advantages of this method in terms of reduced patient discomfort and recovery time.

Further support for the efficacy of cannulas in aesthetic treatments comes from the study demonstrating positive outcomes using both stabilized and non-stabilized hyaluronic acid delivered via cannula [23] which reported significant improvements in skin density and overall skin quality, underscoring the utility of cannulas for precise, minimally invasive delivery of bioactive substances. Our findings align with this evidence, suggesting that cannula-based techniques may offer a superior alternative to traditional methods, especially when combined with multicomponent formulations, which was also confirmed by high-frequency ultrasound proving a reliable method for evaluating outcomes [24,25,26,27].

In summary, skin aging is a multifaceted process driven by both intrinsic cellular mechanisms and extrinsic environmental factors, leading to visible and functional changes in skin structure. Hyaluronic acid (HA) has emerged as a central agent in aesthetic treatments, providing crucial support to the extracellular matrix (ECM), enhancing fibroblast function, and promoting collagen synthesis through the TGF-β/CCN2 pathway. Research underscores that HA-based treatments can effectively counteract age-related skin changes, improving texture, firmness, and hydration while supporting the skin’s natural regenerative capacity. The choice between non-cross-linked and cross-linked HA gels, as well as the use of cannulas for improved patient comfort, highlights the versatility of HA gels in tailored, patient-specific rejuvenation protocols. Continued advancements in HA formulations and application methods will likely enhance therapeutic outcomes, offering patients increasingly effective solutions to maintain youthful, resilient skin. The authors emphasize that future studies should include larger, randomized control groups with extended follow-up durations to validate their findings.

### 7.1. Cannula vs. Needle-Based Approaches 

While this study focused exclusively on cannula-based delivery, it is important to contextualize our findings within the broader landscape of injection techniques. Traditional mesotherapy involves multiple needle injections, which can cause significant discomfort, bruising, and downtime for patients. By contrast, the cannula-based approach used in this study required only one entry point per side of the face, potentially reducing tissue trauma and associated complications. The low incidence of side effects observed in our study (10% bruising, 15% localized swelling) compares favorably with reported rates for needle-based mesotherapy, which can range from 20–30% for bruising [19]. Additionally, the cannula technique allowed for more uniform distribution of the product across the treatment area, potentially contributing to the consistent improvement in skin density observed in our ultrasound measurements. However, needle-based approaches may offer advantages in terms of precise targeting of specific skin layers and may be more suitable for treating fine lines and superficial skin concerns. Future comparative studies are needed to establish clear guidelines for selecting between cannula and needle-based approaches based on patient characteristics and treatment goals.

### 7.2. Bioactive Hydrogels as Multifunctional Therapeutic Platforms in Skin Regeneration

Recent advances in bioactive hydrogel technology have expanded our understanding of their therapeutic potential in tissue regeneration and wound healing. The development of multifunctional hydrogels with targeted biological activities represents a significant advancement in biomaterials science that has implications for both wound management and aesthetic medicine. In this context, two recent studies are particularly relevant. Cheng et al. [28] developed cationic dendritic hydrogels (PHCI) with inherent antibacterial properties designed specifically for infected wound treatment. Their innovative approach uses a novel synthesis strategy without toxic cross-linking agents to create hydrogels that can electrostatically adsorb and kill bacteria while simultaneously promoting hemostasis, modulating inflammation through macrophage phenotype conversion, and accelerating collagen deposition and vascularization. Similarly, Qi et al. [29] created an immunomodulatory hydrogel for diabetic foot ulcers that combines ROS scavenging, oxygen generation, and hyperthermia-induced immunomodulation to transform the wound microenvironment. Both studies exemplify how bioactive hydrogels can be engineered to address multiple pathophysiological processes simultaneously.

Our work extends these principles to the field of aesthetic and regenerative dermatology. The bioactive hydrogel used in our study (Teosyal Redensity 1) combines the structural support of hyaluronic acid with 14 bioactive ingredients. These components potentially address multiple aspects of skin aging, including oxidative stress, decreased fibroblast activity, and impaired extracellular matrix production. While our clinical application differs from infected wound management, the fundamental concept of employing multifunctional bioactive hydrogels to modulate tissue microenvironments is consistent across all three studies. Of particular note is the parallel between collagen deposition induced by Cheng’s antibacterial hydrogel in wound healing and the increased skin density (likely reflecting enhanced collagen organization) observed in our study.

Furthermore, our research advances the existing literature by demonstrating that these sophisticated formulations can be effectively delivered using a minimally invasive cannula-based approach. This represents an important technical innovation that bridges the gap between advanced biomaterial design and practical clinical application. The significant improvement in skin density observed in our ultrasound measurements (92.7%, *p* < 0.001) provides quantifiable evidence of structural enhancement following bioactive hydrogel treatment, suggesting that, similar to wound healing applications, bioactive hydrogels in aesthetic applications may address multiple aspects of skin aging simultaneously.

This integrated approach to skin regeneration represents a promising direction for aesthetic medicine that merges concepts from wound healing, tissue engineering, and dermatological therapies. As the field of bioactive hydrogels continues to evolve, we anticipate further cross-pollination of ideas between wound care and aesthetic applications, ultimately leading to more effective treatments for a range of skin conditions.

## 8. Limitations

This study has several limitations that should be acknowledged. First, the relatively small sample size (n = 20) and the retrospective design limit the generalizability of our findings. A larger, prospective randomized controlled trial would provide stronger evidence for the efficacy of cannula-delivered bioregeneration products. Second, the short follow-up period (3–4 weeks) captures only the immediate effects of the treatment. Future studies should include longer follow-up periods (3–6 months or more) to assess the durability of the observed improvements and determine optimal maintenance protocols. Third, the lack of blinding for both patients and evaluators may have introduced bias in the subjective outcome measures such as GAIS scores and satisfaction surveys. While the ultrasound measurements provide objective data, future studies would benefit from blinded assessments and the use of validated tools such as the FACE-Q for measuring patient satisfaction. Finally, the study did not include a direct comparison with traditional needle-based mesotherapy, which would have provided valuable insights into the relative advantages and disadvantages of the cannula-based approach. Such comparisons should be incorporated into future research designs.

## 9. Conclusions

The use of a cannula for injecting non-cross-linked HA filler provides an effective outcome. The procedure is quick, does not require anesthesia, and involves minimal tissue trauma, eliminating the need for recovery time. The multicomponent bioactive hydrogel used in this procedure significantly improves skin condition in the treated area. The results, particularly in terms of skin density, demonstrate the efficacy of the treatment. Treated areas of the face injected with this non-stabilized HA in the superficial subdermal layer showed a significant increase in skin density three weeks post-procedure. Patient satisfaction surveys also confirmed an improvement in skin condition after the treatment. This work demonstrates the potential of bioactive hydrogels as multifunctional therapeutic platforms that can be effectively delivered via cannula, representing a promising advancement that bridges biomaterial science and aesthetic medicine. While our study establishes this foundation, further research with larger samples and longer follow-up periods will be valuable for confirming these encouraging results.

## Figures and Tables

**Table 1:** Patient-reported outcomes at 3-4 weeks post-treatment (n = 20).

Measure	Category	Result
GAIS Score	Mean score	1.7 ± 0.6
	Worsened (0)	0% (n = 0)
	No change (1)	10% (n = 2)
	Slightly improved (2)	50% (n = 10)
	Noticeably improved (3)	30% (n = 6)
	Significantly improved (4)	10% (n = 2)
Satisfaction	Very satisfied	25% (n = 5)
	Satisfied	60% (n = 12)
	Not satisfied	15% (n = 3)
Recommendation	Would recommend	80% (n = 16)
	Would not recommend	20% (n = 4)

## Data Availability

The data that support the findings of this study are available from the corresponding author upon reasonable request.
